# Lymphatic vessels under pressure

**DOI:** 10.7554/eLife.113304

**Published:** 2026-10-29

**Authors:** Asser F Bedair, Osama F Harraz

**Affiliations:** 1 Larner College of Medicine, University of Vermont Burlington United States

**Keywords:** calcium signalling, lymphatic vessel, lymphatic pressure, lymphatic chronotropy, pressure myography, mechanosensing, Mouse

## Abstract

Lymphatic vessels beat faster as they fill with fluid through a chain of calcium signaling, though the first pressure sensor remains a mystery.

**Related research article** Davis MJ, Kim HJ, Li M, Castorena-Gonzalez JA, Schulz ME, Pea G, Pal S, Domeier TL, Scallan JP, Earley S, Zawieja SD. 2026. Ion channels and GPCR requirements for pressure-induced lymphatic chronotropy: Evidence for a Gαq/11-IP3R1-ANO1 pacemaking axis. *eLife* **14**:RP109466. doi: 10.7554/eLife.109466.

The lymphatic system is the body’s fluid-recovery network. Lymphatic capillaries collect fluid, proteins, immune cells and cellular debris from the interstitial spaces, while larger collecting vessels return this lymph to the bloodstream (Norrmén et al., 2011).

Unlike the blood circulation, however, the mammalian lymphatic network has no central “heart”. Instead, collecting lymphatic vessels act as strings of tiny pumps: rings of lymphatic muscle contract rhythmically, and valves keep lymph moving in the right direction. These miniature pumps also adjust their pace. As a lymphatic vessel fills and pressure rises, rhythmic contractions become more frequent to increase pumping when there is more lymph to move, a response called pressure-induced lymphatic chronotropy (Zawieja et al., 2019). However, the molecular link between pressure and faster rhythm has remained unclear.

Now, in eLife, Michael Davis and colleagues at various research institutes in the United States report on an impressive broad search for this mechanotransduction pathway (Davis et al., 2026). Using a combination of pressure myography to measure muscle contractions, transcriptomics, pharmacology and genetically engineered mice, the team identified the required signaling axis and found – unexpectedly – that many of the obvious candidate sensors are dispensable.

Davis et al. isolated lymphatic vessels from the back legs of mice, which have a large change in their contraction rate when pressure changes (more than 10-fold), making it possible to detect even subtle changes. The researchers began by drawing an analogy between lymphatic vessels and small arteries, which constrict as blood pressure rises and rely on well-characterized signaling to respond to force. They reasoned that if lymphatic muscle used the same machinery, deleting one of these signaling steps should flatten the relationship between pressure and contraction frequency.

The resulting screen was extensive, including many cation channels such as PIEZO1, TRPC6, TRPM4, PKD2, TRPV2, and ENaC, as well as channels involved in pacemaking or membrane excitability, such as NCX1, HCN and Kv7.4. Of sixteen candidates, most proved dispensable for pressure-induced chronotropy, but a few stood out. For example, ANO1 (TMEM16A) is a calcium-activated chloride channel that supports spontaneous contractions (Zawieja et al., 2019). Deleting ANO1 reduced pressure-induced chronotropy. Furthermore, removing the voltage-gated calcium channel Cav1.2, which is required for lymphatic muscle action potentials (To et al., 2020), completely abolished lymphatic contractility.

Considering that ANO1 is not a typical mechanosensor, Davis et al. examined its upstream regulators. Intracellular calcium signals activate ANO1, suggesting that another signaling pathway links pressure to ANO1 activation. Intracellular calcium release events are key triggers of several Ca^2+^-dependent mechanisms and were therefore logical candidates. Indeed, deleting the calcium channel IP_3_R1 impaired pressure-induced chronotropy (Figure 1).

**Figure 1.**
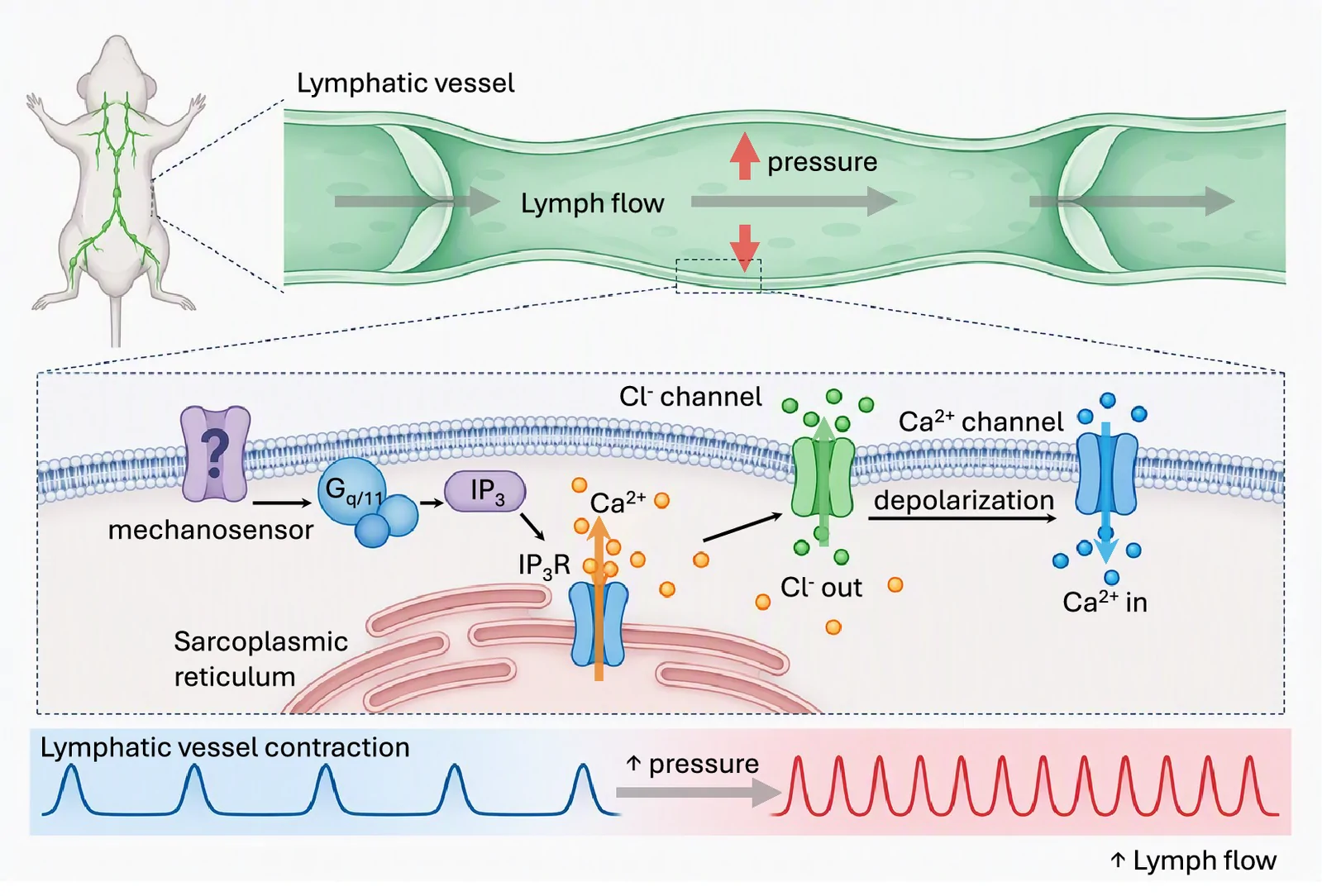
Mechanism of pressure-induced chronotropy in lymphatic vessels. Lymphatic muscle contractions propel lymph through one-way valves that support unidirectional flow (green valve). Increased intraluminal pressure activates an unidentified mechanosensor, which signals through G proteins (G_q/11_, blue circle) to stimulate the production of the signaling molecule IP₃ (purple shape). This, in turn, activates the IP_3_ receptor and triggers calcium (Ca^2+^) release. Subsequently, the calcium-activated chloride channel ANO1 (green) is activated, depolarizing the membrane and therefore activating voltage-gated calcium channels (blue). The following influx of calcium ions increases contraction frequency and promotes lymph flow.

IP_3_ is produced downstream of G proteins – molecular switches that couple cell-surface receptors to intracellular signaling pathways. Deleting either or both components of the G protein complex Gq/11 revealed their critical contribution. Collectively, these results support a relay in which pressure activates Gq/11 signaling, IP_3_ opens IP_3_R1, calcium activates ANO1, and depolarization both activates Cav1.2 and accelerates action-potential firing and contraction.

The pathway becomes less certain at its first mechanosensing step. G-protein-coupled receptors (GPCRs) activate Gq/11. Agonists stimulate GPCRs, while mechanosensitive receptors activate without agonists. Davis et al. identified GPCRs in lymphatic muscle cells and tested seven plausible candidates, including receptors for molecules involved in the occlusion or constriction of blood vessels, such as neuropeptide Y, ET-1, thromboxane and angiotensin II.

While none of the tested receptors was required, these results are informative rather than anticlimactic. One could speculate that multiple receptors share the task, that different receptors operate across varying mechanical ranges, or that the sensor might be an adhesion or orphan GPCR. Even a GPCR-independent route to Gq/11 (for example, through receptor tyrosine kinases) could be a possibility (Garcia-Marcos, 2024). Another important consideration is the residual lymphatic vessel contraction; Gq/11, IP₃R1, or ANO1 disruption greatly suppressed chronotropy at lower pressures, but what additional mechanisms operate at higher pressures, where contraction frequency continues to increase despite disrupted calcium channels? Perhaps, lymphatic muscle cells retain a basal pacemaker through the identified axis, and additional unidentified mechanisms provide routes to faster firing at higher pressures.

Davis et al. have placed the signaling pathway involving Gq/11, IP₃R and ANO1 at the center of pressure-induced lymphatic chronotropy, while ruling out a long list of alternative candidates. Important questions remain, however. Understanding how lymphatic vessels sense pressure could reveal therapeutic targets for restoring lymphatic function in diseases such as lymphedema, in which fluid accumulates in tissues and causes swelling (Olszewski, 2008). The pathway driving the lymphatic heartbeat is coming into focus, but the molecule that first feels the pressure remains hidden.
